# Red blood cell distribution width and platelet counts are independent prognostic factors and improve the predictive ability of IPI score in diffuse large B-cell lymphoma patients

**DOI:** 10.1186/s12885-019-6281-1

**Published:** 2019-11-11

**Authors:** Manman Li, Hailong Xia, Huimin Zheng, Yafeng Li, Jun Liu, Linhui Hu, Jingrong Li, Yangyang Ding, Lianfang Pu, Qianle Gui, Yijie Zheng, Zhimin Zhai, Shudao Xiong

**Affiliations:** 1 Department of Hematology/Hematological Lab, The Second Hospital of Anhui Medical University, Hefei, Anhui Province 230601 People’s Republic of China; 2 Hematology Research Center, Anhui Medical University, Hefei, 230601 People’s Republic of China; 3 Department of Hematology, Chaohu Hospital of Anhui Medical University, Chaohu, 238000 People’s Republic of China; 4 Department of Hematology, The First Hospital of Anhui Medical University, Hefei, 230000 People’s Republic of China; 5 Department of Emergency, The Second Hospital of Anhui Medical university, Hefei, 230601 Anhui Province People’s Republic of China; 6 Department of Hematology, The Third People’s Hospital of Bengbu, Bengbu, 233000 People’s Republic of China; 7 Department of Immunology and Key Laboratory of Molecular Medicine of Ministry Education, Shanghai Medical College, Fudan University, Shanghai, 200032 People’s Republic of China

**Keywords:** Prognosis, Diffuse large B-cell lymphoma (DLBCL), Red blood cell distribution width (RDW), Platelet count (PLT)

## Abstract

**Background:**

Elevated red blood cell distribution width (RDW) and decreased platelet count (PLT) can be clinically relevant to the prognosis in cancer patients. However, their prognostic values in patients with diffuse large B-cell lymphoma (DLBCL) need to be further explored.

**Methods:**

Healthy donors (*n* = 130) and patients with DLBCL (*n* = 349) were included and evaluated retrospectively in this study. The prognostic influence of clinical and pathological factors including RDW and PLT on overall survival (OS) and progression-free survival (PFS) were studied by Kaplan-Meier curves. To evaluate the independent prognostic relevance of RDW and PLT, univariate and multivariate Cox proportional hazards regression models were applied. The adjusted IPI model was established based on the results of multivariate analysis, and verified by Harrell’s C statistical analysis.

**Results:**

Kaplan-Meier curves indicated that an elevated RDW value and thrombocytopenia are poor factors for OS (*P* < 0.001, *P* = 0.006) and PFS (*P* = 0.003, *P* < 0.001) in DLBCL patients. Multivariate analysis confirmed that elevated RDW value (HR = 2.026, 95%CI = 1.263–3.250, *P* = 0.003) and decreased PLT count (HR =1.749, 95%CI = 1.010–3.028, *P* = 0.046) were both independent prognostic factors. The c-index of IPI and NCCN-IPI were increased when RDW level and PLT were supplemented in our cohort.

**Conclusions:**

Our study shows that elevated RDW level and decreased PLT are independent poor prognostic factors in newly diagnosed DLBCL patients. Adding RDW and PLT to the IPI score may improve its predictive ability, and the adjusted IPI may be more powerful in predicting the survival of DLBCL patients in the rituximab era.

## Background

Diffuse large B-cell lymphoma (DLBCL) is the most common type of aggressive lymphomas in hematological malignancies. In the newly diagnosed cases of non-Hodgkin’s lymphomas (NHL), about 30 to 40% are DLBCL [1]. Although the survival of DLBCL patients has been greatly improved with the administration of CHOP chemotherapies (cyclophosphamide, doxorubicin, vincristine and prednisolone) with rituximab, there are still about 10–15% of patients suffered from primary refractory disease, and about 20–30% relapsed [2].

DLBCL is a highly heterogeneous disease, and a variety of factors affect its prognostic assessment. The clinicians characterize prognosis in aggressive NHL (predict the risk of disease progression, recurrence and mortality/death) based on clinical risk factors, and the related predictive index. The most commonly used clinical prognostic marker during the pre-rituximab era was the International Prognostic Index (IPI) [3], which contains five features: age, tumor stage, serum lactate dehydrogenase (LDH) concentration, performance status and the number of extranodal disease sites. In addition, with widespread applications of rituximab, the NCCN-IPI for DLBCL has been proposed and adopted, which is more efficient in predicting the survival of DLBCL patients in the rituximab era [4]. However, the prognosis of patients with poor outcomes has not been fully elucidated, so some clinical factors that provide prognostic information are needed to better assess the prognosis of patients with DLBCL.

Recently, some of the prognostic significance of biochemical markers, molecular genetic markers and immunohistochemical characteristics have gradually been identified [2]. But these markers are usually associated with high cost, laborious laboratory tasks, high technical skill requirements and time-consuming procedures, which are not feasible to conduct in most laboratories. Thus, identifying cheaper and easily available prognostic surrogate markers may contribute greatly to improve the risk assessment for patients with various cancers including DLBCL.

It is well-known that tumor-associated inflammatory response can promote tumorigenesis and progression [5]. Accumulating studies have confirmed that the relationship between inflammation-related clinical parameters are related to tumor biology and prognosis. These clinical parameters include red blood cell distribution width (RDW) [6], neutrophil/lymphocyte ratio (NLR) [7], lymphocyte/monocyte ratio (LMR) [8] and PLT [9]. However, there are very few reports on the prognostic value of RDW and PLT in patients with DLBCL. Neither of the recently developed R-IPI nor NCCN-IPI prognostic score including the two factors and the role of RDW and PLT count in their scores, also, their roles as independent prognostic factors in DLBCL has never been fully explored. Therefore, this study seeks to evaluate the prognostic significance of RDW and PLT in a large cohort of DLBCL patients, and to test whether they can significantly improve the predictive power of the IPI score in DLBCL patients.

## Methods

### Patients and healthy donor participants

A total of 349 patients with DLBCL were analyzed for retrospective studies, they were diagnosed according to the 2016 World Health Organization criteria [10] at the First and Second Affiliated Hospitals of Anhui Medical University, from July 2006 to April 2017, respectively. The study ethic approval was granted from the local ethical committee of Anhui Medical University, and was performed in accordance with the principles of the Declaration of Helsinki.

Patients were excluded if they were found to be HIV-positive. Other exclusion criteria included transformed indolent lymphoma and primary DLBCL of the central nervous systems (CNS). The rest of DLBCL patients (*n* = 349) were treated with standard CHOP chemotherapy with or without rituximab. In addition, age and sex-matched 130 healthy donors (HDs) from the Second Affiliated Hospital of Anhui Medical University were recruited as normal control group.

Of the 349 DLBCL patients, we randomly selected 200 patients as the training set, while the remaining patients were assigned to the testing set (*n* = 149) [11]. The demographic characteristics, clinical features and laboratory parameters were obtained from the patient’s medical records from both institutions. Retrieved clinical-pathological parameters included gender, age, lactate dehydrogenase (LDH) level, Ann Arbor stage, number of extra nodal sites involvement, Eastern Cooperative Oncology Group performance status (ECOG PS), B symptoms, physical examinations, computed tomography (CT) scans of the thorax, abdomen and pelvic cavity, along with whole-body positron emission tomography (PET/CT) scans and the process of treatment. Laboratory parameters such as complete blood count, biochemical profiles were collected at the time of diagnosis. The date of death was obtained from the clinical records or by telephone calls to their relatives.

### Statistical analyses

The primary end point of the study was overall survival (OS), and the secondary end point was progression-free survival (PFS). OS was defined as the time from the date of diagnosis to the date of death due to any causes within the follow-up period or to the date of the last follow-up. PFS was defined as the time from the date of diagnosis to the date of tumor progression, recurrence or death due to any causes.

Student’s t-test was used to test the differences between the two groups for quantitative normally distributed variables and the Mann-Whitney U test was used for non-parametric variables. Correlation was assessed using Spearman rank test. The optimal cutoff value of training set (*n* = 200) for RDW and PLT were determined by applying receiver operating characteristic curve (ROC) analysis based on previously published reports [12]. Pearson’s Chi-square or Fisher’s exact test was used to assess the associations between RDW, PLT and clinical-pathological parameters. The associations between RDW and PLT levels with OS and PFS respectively were estimated by Kaplan–Meier curves; and log-rank test was used for comparison between different groups.

Multivariate analyses of independent clinical factors for OS and PFS were conducted using the Cox analysis with the forward selection method. Hazard ratios (HRs) estimated from the Cox analysis were reported as relative risks with corresponding 95% confidence intervals (CIs). C-index was calculated using the individual IPI value followed by the addition of the RDW and PLT levels [13]. All statistical analyses were performed using the Statistical Package for the Social Sciences (SPSS 19.0, USA) and R version 3.4.3 (https://www.r-project.org/). *P* < 0.05 was considered statistically significant and *P*-values were two-tailed.

## Results

### The characteristics of DLBCL patients and healthy donors

Healthy donors (*n* = 130) and patients with DLBCL (*n* = 349) confirmed by previous histopathological analysis were included in the study. A full list of clinical characteristics of healthy donors and DLBCL patients were listed in Additional file 1: Table S1. It showed that DLBCL patients and healthy donors had similar age, gender, white blood cell count (WBC), absolute neutrophil count (ANC), platelet count (PLT) and albumin/globulin ratio (AGR). However, the absolute monocyte count (AMC) and RDW in DLBCL patients were significantly higher than that in healthy controls; and the absolute lymphocyte count (ALC), hemoglobin (Hb), albumin (ALB) and globulin (GLB) in DLBCL patients were significantly lower than healthy donors. There were 174 patients (49.9%) treated with R-CHOP, and 175 patients (50.1%) treated with CHOP only. The subgroups of patients’ Ann Arbor tumor stage were 89 (25.5%) in stage I, 86 (24.6%) in stage II, 61 (17.5%) in stage III and 113 (32.4%) in stage IV. There were no statistical differences in the age, gender, and other clinicopathological parameters between the training set and the testing set (Additional file 1: Table S2).

### Cut-off values of RDW and PLT in DLBCL patients

RDW, PLT and Hb are three common parameters in routine blood test. Using ROC analysis and calculating the Youden index (specificity+sensitivity–1), the optimal cutoff values chosen for RDW and PLT were 14.35% and 126.5 × 10^9^/L respectively in the training set (Additional file 1: Figure S1). However, there are gender differences in the definition of anemia. According to the guidelines of the World Health Organization, anemia in male patients is defined as hemoglobin (Hb) < 13 g/dL, and female patients have Hb < 12 g/dL. The cutoff values were applied to the whole cohort, DLBCL patients were then classified into high-level and low-level groups, where 93 (26.64%) patients fell in the high RDW group, 44 (14.43%) patients in low PLT group, and 187 patients with anemia.

### Association of RDW, PLT and Hb with other clinical- pathological factors

Linear correlation analysis showed that higher RDW level was associated with higher NLR, lower ALB and lower Hb; while lower PLT correlated directly with lower WBC, but did not correlate with NLR, ALB or Hb (Additional file 1; Figure S2).

Further analysis showed that, the value of RDW > 14.35% significantly correlated with a poorer ECOG-PS (*P* < 0.001), more extranodal sites of disease (*P* = 0.002), presence of B symptoms (*P* = 0.011), bone marrow involvement (*P* = 0.007), higher Ann Arbor stage (*P* < 0.001), higher LDH level (*P* < 0.001) and higher IPI score (*P* < 0.001). However, we found no statistical significance between age and gender with RDW level. There were also significant correlations between patients with PLT≦126.5 × 10^9^/L and higher Ann Arbor stage (*P* = 0.003); more extranodal sites of disease (*P* = 0.021); higher LDH level (*P* = 0.013) and presence of B symptoms (*P* = 0.033). There were no statistical correlations between low PLT with age, gender and bone marrow involvement. In addition, ECOG PS (*P* = 0.096) and IPI score (*P* = 0.061) had only borderline significance (Table 1). Because of the correlation between RDW and Hb, we further analyzed the correlation between Hb level and clinical parameters. Lower Hb level was significantly associated with higher NLR (*r* = 0.253, *P* < 0.001) and higher ALB (*r* = 0.519, *P* < 0.001), but not correlated with WBC or NLR (Additional file 1: Figure S3). Overall, Hb level was significantly associated with age, gender, B symptoms, clinical disease stage, serum LDH level, ECOG-PS, extranodal sites of disease and IPI score, but it was not associated with bone barrow involvement (Table 1).

**Table 1:** Patient’s baseline characteristics at diagnosis of all patients

Characteristics	RDW(%)		*P* value	PLT(×10^9^/L)		*P* value	HB(g/dL)		*P* value
	> 14.35(*n* = 93,%)	≦14.35(*n* = 256,%)		> 126.5(*n* = 305,%)	≦126.5(*n* = 44,%)		low(*n* = 187,%)	high(*n* = 162,%)	
Age > 60	38(40.86)	108(42.19)	0.824	126(41.31)	20(45.45)	0.602	88(47.06)	58(35.80)	0.034
Gender (male)	43(46.23)	148(57.81)	0.055	168(55.08)	23(52.27)	0.726	91(48.66)	100(61.73)	0.014
B symptoms (present)	37(39.78)	66(25.78)	0.011	84(27.54)	19(43.18)	0.033	73(39.04)	30(18.52)	< 0.001
Ann Arbor stage III/IV	63(67.74)	111(43.36)	< 0.001	143(46.89)	31(70.45)	0.003	117(62.57)	57(35.19)	< 0.001
ECOG PS≧2	51(54.83)	49(19.14)	< 0.001	75(24.59)	16(36.36)	0.096	75(40.11)	16(9.88)	< 0.001
Serum LDH level≧246u/l	58(62.37)	88(34.38)	< 0.001	122(40.00)	24(54.54)	0.013	97(51.87)	49(30.25)	< 0.001
Extranodal sites≧2	34(36.56)	52(20.31)	0.002	69(22.62)	17(38.64)	0.021	57(30.48)	29(17.90)	0.007
BM involvement	9(9.68)	6(2.34)	0.007	11(3.61)	4(9.09)	0.201	11(5.88)	4(2.47)	0.192
IPI > 2	44(47.31)	57(22.27)	< 0.001	83(27.21)	18(40.91)	0.061	74(39.57)	27(16.67)	< 0.001

### Levels of RDW, PLT, Hb at diagnosis and clinical outcomes

**Fig. 1:**
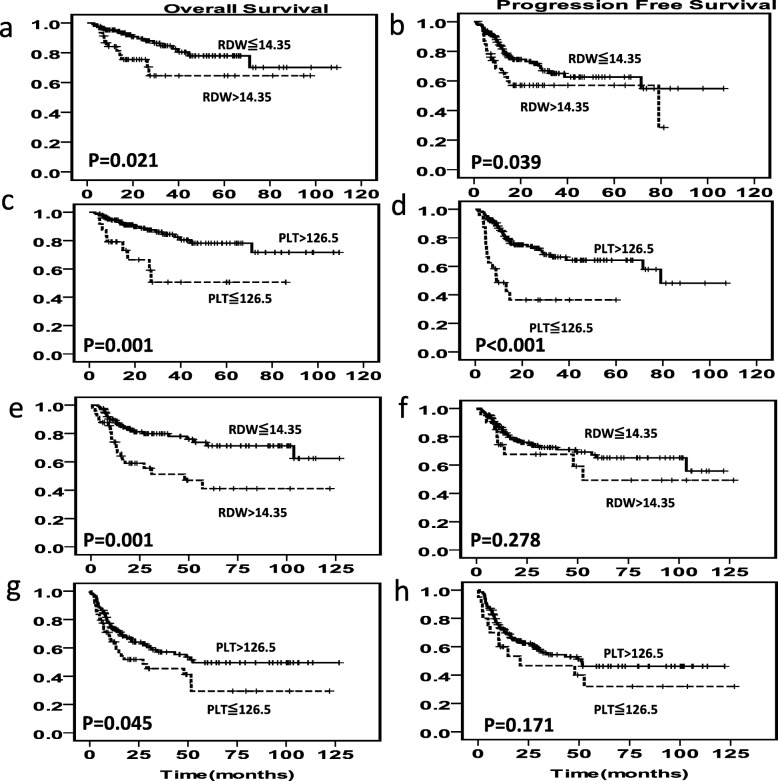
Survival curves according to RDW and PLT levels in the R-CHOP and CHOP cohort. OS and PFS according to RDW (**a**, **b**) and PLT (**c**, **d**) levels in the R-CHOP cohort. OS and PFS according to RDW (**e**, **f**) and PLT (**g**, **h**) levels in the CHOP cohort

The median follow-up time for our study was 21.3 months (range: 0.80–126.93). During follow-up, a total of 134 (38.4%) patients presented with disease recurrence, disease progression or death, of which 79 (22.6%) died. In the training set, the survival rate was significantly worse in patients with higher RDW than in patients with lower RDW (5-year OS: 43%vs 69%; 5-year PFS: 29%vs 53%) (Additional file 1: Figure S4a,S4b). Also, patients with lower PLT showed significantly worse PFS than the patients with higher levels (5-year PFS: 30% vs 49%) (Additional file 1: Figure S4d), but the overall survival was not significantly different (*P* = 0.074) (Additional file 1: Figure S4c). Similar results were observed in the testing set and the whole cohort set (Additional file 1: Figure S4e-4 l). In order to explore whether different chemotherapy regimens affect the evaluation efficacy of the level of RDW and PLT, we divided the patients into two groups, one group treated with R-CHOP regimen and the other group treated with CHOP regimen. Kaplan-Meier analysis showed poor OS and PFS in patients with high RDW (*P* = 0.021 for OS and *P* = 0.039 for PFS) and low PLT (*P* = 0.001 for OS, *P* < 0.001 for PFS) levels in the R-CHOP cohort. Patients with higher RDW and lower PLT in CHOP treated cohort had poorer OS (*P* = 0.001 for RDW, *P* = 0.045 for PLT), but the results of PFS were not statistically significant (Fig. 1). Next, we analyzed the correlation between Hb level and other clinical-pathological parameters. We found that anaemic patients had poorer OS in the training set and CHOP cohort, and poorer OS and PFS in the overall set. (Additional file 1: Figure S5).

**Fig. 2:**
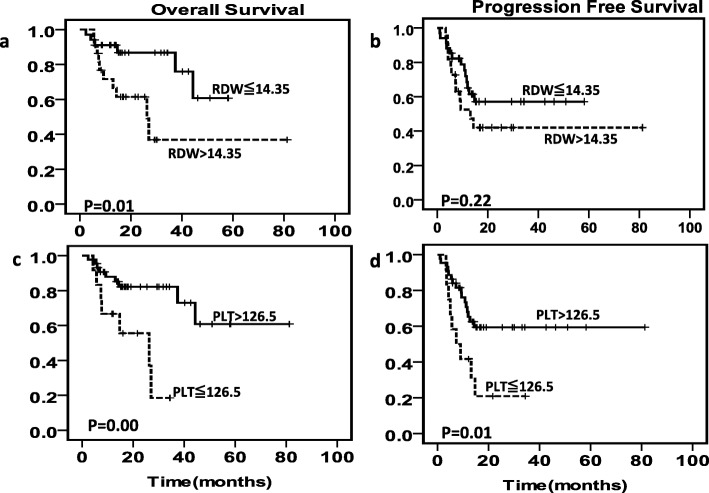
Survival curves according to RDW and PLT levels in IPI score 3–5 in R-CHOP cohort. (1) OS(**a**) and PFS(**b**) according to RDW levels in IPI score 3–5 in R-CHOP cohort. (2) OS(**c**) and PFS(**d**) according to PLT counts in IPI score 3–5 in R-CHOP cohort

We further assessed the prognostic value of RDW, PLT and Hb in the IPI subgroup. The Kaplan-Meier analysis showed that the RDW, PLT and Hb levels may not distinguish those with favorable outcomes from those with adverse outcomes for patients with IPI score of 0–2 (data not shown). However, in patients with IPI scores 3–5, the RDW and PLT levels, but not Hb level (data not shown) were able to further risk-stratify patients into high-risk and low-risk groups. In R-CHOP cohort, the patients with lower PLT had significantly poorer OS (*P* = 0.003) and PFS (*P* = 0.013); and in higher level of RDW patients, OS (*P* = 0.014) was significantly reduced (Fig. 2); the whole cohort and CHOP cohort also showed similar results (Additional file 1: Figure S6).

### High RDW, low PLT and Hb at diagnosis as poor prognostic factors

To investigate the association between RDW and PLT and Hb levels with patients’ clinical outcomes, we performed the Cox proportional risk model. Table 2 and Table 3 summarized the results of the univariate and multivariate analysis for factors influencing OS and PFS in all DLBCL patients. The univariate Cox proportional analysis revealed that old age, advanced Ann Arbor stage, poor ECOG PS, elevated LDH, B symptoms, more extranodal sites of disease, higher IPI score, bone marrow involvement, lower Hb level, higher RDW and lower PLT were all predictors of DLBCL patients for OS and PFS (Table 2). To explore whether RDW and PLT were independent prognostic factors of DLBCL patients, we performed a multivariate analysis, including age, advanced Ann Arbor stage, ECOG PS, LDH, extranodal sites, B symptoms, IPI score, bone marrow involvement, lower Hb level, RDW and PLT. Interestingly, our results showed that older age (*P* < 0.001), advanced Ann Arbor stage (*P* = 0.037), higher RDW (*P* = 0.003) and lower PLT (*P* = 0.046) were independent prognostic factors for OS. On the other hand, for PFS, only older age (*P* < 0.001), advanced Ann Arbor stage (*P* = 0.002) and lower PLT(*P* = 0.002) were independent prognostic factors (Table 3). But the ECOG PS, LDH, extranodal sites, B symptoms, IPI, bone marrow involvement and lower Hb level were not independent prognostic factors for OS and PFS in our study for DLBCL patients.

**Table 2:** Univariate analysis of clinicopathological parameters for the prediction of OS and PFS in DLBCL patients(*n* = 349)

Parameter	Number	%	Overall survival			Progression-free survival		
			HR	95%CI	*P* value	HR	95%CI	*P* value
Gender(male)	191	54.73	0.951	0.610–1.484	0.825	1.044	0.744–1.465	0.804
age > 60	146	41.83	3.437	2.146–5.504	< 0.001	2.42	1.715–3.414	< 0.001
PLT≦126.5(×10^9^/L)	44	12.61	2.100	1.227–3.594	0.007	2.164	1.409–3.324	< 0.001
RDW > 14.35%	93	26.65	2.652	1.695–4.151	< 0.001	1.706	1.191–2.443	0.004
Low Hb level	187	53.58	1.817	1.148–2.876	0.011	1.479	1.047–2.089	0.027
B symptoms(present)	103	29.51	2.142	1.365–3.360	0.001	1.613	1.131–2.300	0.008
Ann Arbor stage III/IV	174	49.86	2.985	1.844–4.832	< 0.001	2.462	1.727–3.510	< 0.001
ECOG PS > 1	91	26.07	2.685	1.718–4.198	< 0.001	1.908	1.337–2.724	< 0.001
LDH > normal	146	41.83	2.159	1.381–3.375	0.001	1.844	1.312–2.590	< 0.001
Extranodal sites> 1	86	24.64	2.457	1.560–3.870	< 0.001	2.313	1.621–3.301	< 0.001
BM nvolvement	15	4.30	2.269	1.043–4.936	0.039	1.494	0.731–3.055	0.271
IPI > 2	101	28.94	4.097	2.616–6.417	< 0.001	2.727	1.932–3.849	< 0.001

**Table 3:** Multivariate analysis of clinicopathological parameters for the prediction of OS and PFS in DLBCL patients(*n* = 349)

Parameter	Overall survival		*P* value	Score	Progression-free survival			*P* value
	HR	95%CI			HR	95%CI		
age > 60	3.012	1.817–4.994	0.000	2	2.199	1.529–3.163		0.000
Ann Arbor stage III/IV	1.887	1.040–3.423	0.037	1	1.936	1.263–2.966		0.002
PLT≦126.5(×10^9^/L)	1.749	1.010–3.028	0.046	1	1.963	1.274–3.024		0.002
IPI > 2	1.771	0.984–3.187	0.057		1.499	0.977–2.299		0.064
RDW > 14.35%	2.026	1.263–3.250	0.003	1				0.293

We further performed univariate and multivariate analysis by applying the above indicators to the R-CHOP and CHOP cohorts. Bone marrow involvement in the univariate analysis was not statistically significant and the number of patients involved in bone marrow was small, hence, it was excluded from the multivariate analysis (Additional file 1: Table S3 and Table 4). Surprisingly, we found that elevated RDW was an independent prognostic factor (*P* = 0.012) in CHOP cohort, and depressed PLT was an independent prognostic factor (*P* = 0.003) in R-CHOP cohort for OS. However, RDW was not an independent prognostic factor for PFS either in R-CHOP cohort or in CHOP cohort, whereas PLT was an independent prognostic factor (*P* = 0.003) in R-CHOP cohort but not in CHOP cohort (Table 4).

**Table 4:** Multivariate analysis for OS and PFS of patients treated with or without rituximab

Parameter	Overall survival		*P* value	Progression-free survival		*P* value
	HR	95%CI		HR	95%CI	
CHOP cohort(*n* = 175)						
RDW > 14.35%	2.123	1.183–3.812	0.012			0.502
age > 60	3.449	1.777–6.692	0.000	3.434	2.093–5.634	0.000
Ann Arbor stage III/IV	3.155	1.702–5.847	0.000	2.814	1.785–4.436	0.000
R-CHOP cohort(*n* = 174)						
PLT≦126.5(×10^9^/L)	3.344	1.491–7.504	0.003	3.076	1.653–5.723	0.000
age > 60	2.344	1.090–5.039	0.029			0.333
IPI > 2	2.304	1.061–5.002	0.035			0.322
Extranodal sites> 1			0.772	2.347	1.371–4.020	0.002

### Development of a modified IPI by adding both RDW and PLT

From multivariate analysis, there were clearly four independent prognostic factors for OS in the whole cohort. We then used the four clinical parameters to construct a new adjusted IPI model, age > 60 equaled to two points; RDW > 14.35%, PLT≦126.5(× 109/L) and Ann Arbor stage III/IV equaled to one point respectively [14]. Three risk categories were generated: low (0–1 points), intermediate (2–3 points) and high (4–5 points).

**Fig. 3:**
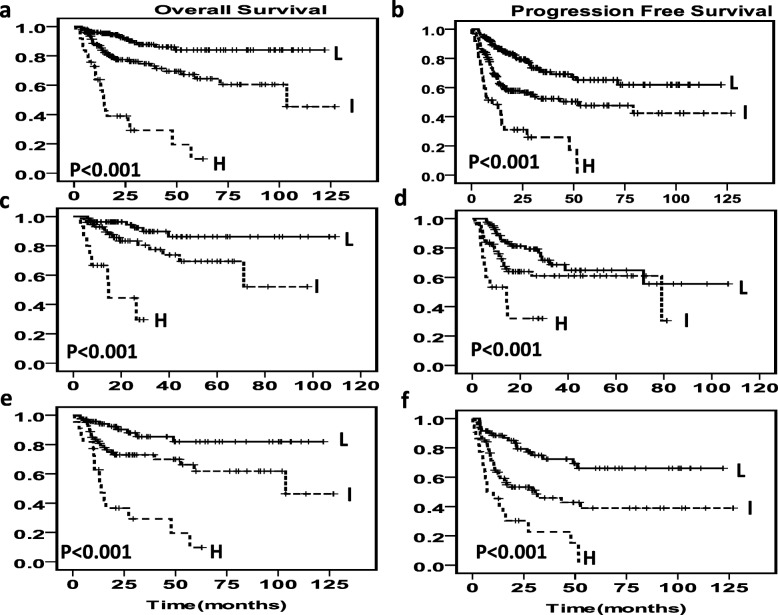
Adjusted IPI survival curves based on the addition of RDW and PLT. (1) Survival curves for OS (**a**) and PFS (**b**) according to adjusted IPI of adding RDW and PLT for risk stratification in the whole cohort. (2) Survival curves for OS (**c**) and PFS (**d**) according to adjusted IPI of adding RDW and PLT for risk stratification in R-CHOP cohort. (3) Survival curves for OS (**e**) and PFS (**f**) according to adjusted IPI of adding RDW and PLT for risk stratification in CHOP cohort

Based on the risk stratification model, the results showed that patients assigned to the low-risk group had good outcomes (5-year OS: 83%, 5-year PFS: 62%) and high-risk patients had very poor outcomes (5-year OS: 9%, 5-year PFS: 0%, Fig. 3a,b) in all patients cohort. Similar results were observed in the R-CHOP (*n* = 174) cohort (Fig. 3c, d) and CHOP cohort (*n* = 175) (Fig. 3e, f). To strengthen the results from the multivariate analysis, we conducted a Harrell’s C statistics analysis. The c-index of the IPI prognostic model for OS was 0.744 for patients treated with CHOP, 0.709 for patients treated with R-CHOP, 0.725 for all DLBCL patients, and 0.763, 0.718, 0,743 in NCCN-IPI prognostic model. When the factors of RDW and PLT values were added, the predictive power was increased in both IPI and NCCN-IPI prognostic model. And the c-index of the adjusted IPI in the three cohorts was 0.753, 0.732 and 0.748 (Table 5).

**Table 5:** Harrell’s C statistic for discriminatory values on survival

Parameter	CHOP cohort	R-CHOP cohort	All Patients
IPI	0.744	0.709	0.725
NCCN-IPI	0.763	0.718	0.743
PLT	0.527	0.611	0.557
RDW	0.613	0.618	0.616
IPI + RDW	0.750	0.710	0.728
NCCN-IPI + RDW	0.769	0.718	0.743
IPI + PLT	0.745	0.726	0.729
NCCN-IPI + PLT	0.762	0.731	0.746
IPI + RDW + PLT	0.751	0.733	0.731
NCCN-IPI + RDW + PLT	0.767	0.732	0.747
adjusted IPI	0.753	0.732	0.748

## Discussion

Our results indicate clearly that RDW and PLT levels are independent risk factors for patients with DLBCL. In addition, for patients who are treated with R-CHOP like regimens, PLT is a significant prognostic factor for OS. Similarly, for patients who are treated with CHOP like regimens, RDW is a more important prognostic factor. In addition, we first discovered that the combination of RDW and PLT with IPI can further improve the prognostic value and clinical significance of IPI and NCCN-IPI.

As a commonly used indicator for tumor-associated inflammatory responses, RDW has been widely studied and has been proved to be associated with the prognosis of a variety of diseases [6]. There is growing evidence demonstrating elevated RDW as a prognostic factor in various malignancies, such as lung cancer [15], prostate cancer [16], chronic lymphocytic leukemia [17], ovarian cancer [18], hilar cholangiocarcinoma [19] and Esophageal carcinoma [20]. Some studies have confirmed the close relationship between high RDW and cancer stage [21, 22].

The exact mechanism for the elevation of RDW in DLBCL patients is not clear. Lippi et al. [6] demonstrated that short telomeres length, oxidative stress, inflammation, erythrocyte fragmentation, poor nutritional status, hypertension, dyslipidemia and abnormality of erythropoietin function may be the causes. These factors may lead to a profound deregulation of erythrocyte homeostasis including impaired erythropoiesis, abnormal erythrocyte metabolism and survival which resulted in elevated RDW. Lymphoma is a malignant tumor that originates from the lymphatic hematopoietic system. Patients with malignant diseases often have chronic inflammation and poor nutritional status. Some studies reported that elevated RDW was correlated with higher IL-6 [23] and erythrocyte sedimentation rate (ESR), as well as high-sensitivities of C-reactive protein (CRP), leukocytes, neutrophils, fibrinogen, and lower Hb [24, 25]. Further research supports RDW being associated with erythropoietin (EPO) [26], ALB [27], iron, folate and vitamin B12 [28]. However, in our study, elevated RDW was associated with poorer ECOG-PS, more extranodal sites of disease, B symptoms, higher Ann Arbor stage, higher LDH, higher IPI, higher NLR, lower ALB and lower Hb. In consideration of previous studies and our findings, it is rational to conclude that RDW is associated with tumor burden, chronic inflammation and malnutrition in DLBCL patients. All these factors are well-known to lead to poor prognosis in cancer patients. Cancer related inflammation is considered a landmark feature of cancer development and progression [5]. Inflammatory mediators and cytokines are important components of the tumor microenvironment, which sustains the progression of the tumor [29]. Poor nutritional status was another hallmark of cancer [30]. Inflammation and malnutrition might damage erythropoiesis, thus resulting in an increased RDW. In 2018, Zhou et al. analyzed the relationship between RDW and normal erythropoiesis/megakaryocytopoiesis in multiple myeloma patients at diagnosis and their study demonstrated the usefulness of RDW as an indicator for bone marrow hematopoiesis [31]. In our study, patients with high RDW and anemia had high bone marrow involvement rate (RDW:*P* = 0.007, anemia: *P* = 0.192), which may be due to the influence of bone marrow microenvironment on hematopoiesis. However, in our study, Hb levels and bone marrow involvement were statistically significant in univariate COX analysis in the overall set, but not in multivariate analysis, and this result may be related to the small number of patients with bone marrow involvement. At present, whether anemia and bone marrow involvement are independent prognostic factors for patients with DLBCL have not reached a unified conclusion [3, 32–34]. It may be related to the difference of patients in the study and further studies with a large cohort is needed to improve on the statistics.

How PLT level affects the outcome of DLBCL remains speculative, which probably attributes to the reduction of platelets in lymphoma patients. In our study, patients with low PLT levels had a high rate of bone marrow involvement, but this was not statistically significant(*P* = 0.201). This suggests that thrombocytopenia may be affected by a variety of factors. The reduction in platelets count can be caused by several factors such as drug, malignant infiltration of bone marrow, consumptive infection, splenic sequestration, pre-existing viral hepatitis, myelodysplasia and immune-mediated destruction, as reported by Liebman H [35]. Some studies reported that lymphoma patients with thrombocytopenia presented poor survival if the patients had bone marrow involvement [36, 37]. However, it has been found that the prognostic effect of platelet counts was not consistent. In solid tumors, e.g., the elevated platelet count is poor prognostic factor and plays an important role in the progression and metastasis. The potential mechanisms include protecting circulating tumor cells from attacking host’s immune system as well as supporting proliferation of tumor cells [38]. But, contradictorily, in many hematological diseases, patients with low PLT have a poor prognosis, such as Ph-like acute lymphoblastic leukemia [39], hemophagocytic lymphohistiocytosis (HLH) [40], primary plasma cell leukemia (pPCL) [41] and DLBCL [42, 43]. Our data demonstrated that PLT≦126.5 × 10^9^/L was associated with higher Ann Arbor stage, more extranodal sites, higher LDH, lower WBC. These results suggested that patients with low levels of PLT may have a higher tumor burden; in addition, low levels of PLT may be associated with the expansion of myeloid lines such as myeloid derived suppressor cells (MDSCs), macrophages and dendritic cells (DCs), as well as the reduction of mature red blood cells and platelets [44]. But the exact function of platelets in the tumor microenvironment remains unclear. In our study, we propose that immune disorders, high tumor burden, bone marrow involvement and low level of neutrophils are associated with poor prognosis in patients with thrombocytopenia in DLBCL.

Previous studies showed that RDW and PLT are independent predictive factors for survival in DLBCL [27, 42, 43, 45], but their sample sizes were small. However, no study has further analyzed the c-index that is important to calculate the discriminative degree between the predicted value and the value of the COX model in survival analysis [46], nor evaluated the significance of RDW and PLT for IPI. Therefore, our study further expanded the sample size, and validated the prognostic significance of RDW and PLT for patients with DLBCL, and to construct a simpler and more useful prognostic model for DLBCL patients.

Based on the results of the multivariate analysis, we have constructed a new prognostic model which includes four independent prognostic factors: age > 60 years, Ann Arbor stage > 2, PLT≦126.5 × 10^9^/L and RDW > 14.35%. The adjusted IPI is easy to use and effectively divides patients with DLBCL into three risk groups. And then, to confirm the prognostic value of RDW and PLT, we conducted a Harrell’s C statistics analysis for OS. Our results suggested that combined RDW and PLT with the IPI score have a good prognostic value for patients with DLBCL, especially in patients with CHOP regimen chemotherapy. Adding RDW and PLT to the well-established prognostic models such as the IPI score might improve their predictive ability.

The cutoff values of the parameters were obtained according to the Youden index from training set, and then it was used to measure the impact of RDW and PLT on OS and PFS in DLBCL for training set, testing set, whole patients set, CHOP cohort and R-CHOP cohort. The inclusion of validation steps in this study has greatly increased the reliability of our data, and the results demonstrated that our new prognostic models may be generally applicable to DLBCL patients.

Although our results are consistent with those previously reported, our study has several limitations. Firstly, as a retrospective study with a relatively small number of patients, a regional or phenotypical selection bias is inevitable. Secondly, due to the widespread use of rituximab, we can expand the sample size of the R-CHOP group and further explore the effects of RDW and PLT on NCCN-IPI. Thirdly, few patients had bone marrow involvement. Despite these limitations, our research provides new ideas for establishing a simpler, more practical, and accurate risk model for the prognosis of patients with DLBCL.

## Conclusions

In conclusion, RDW and PLT levels are simple and useful independent prognostic factors in DLBCL patients. The adjusted IPI by adding both RDW and PLT is an effective and valuable risk stratification model for DLBCL patients, and may be more potential to predict the survival of DLBCL patients in the rituximab era.

## Supplementary information

**Additional file 1: Figure S1.** ROC curves analysis for RDW(a) and PLT(b) in the training set (N=200) of patients with DLBCL. **Figure S2.** Correlation between RDW, PLT and WBC, NLR, ALB, HB levels in all patients with DLBCL. Correlation between RDW and WBC(a), NLR(b), ALB(c) and HB(d) levels in all patients with DLBCL. (2)Correlation between PLT and WBC(e), NLR(f), ALB(g) and HB(h) levels in all patients with DLBCL. **Figure S3.** Correlation between HB and WBC(a), NLR(b) and ALB(c) levels in all patients with DLBCL. **Figure S4.** Survival curves according to RDW and PLT levels in the training, overall and testing set. (1)OS(a,c) and PFS(b,d) according to the RDW and PLT levels in the training set. (2)OS(e,g) and PFS(f,h) according to the RDW and PLT levels in the overall set. (3)OS(i,k) and PFS(j,l) according to the RDW and PLT levels in the testing set. **Figure S5.** Kaplan–Meier curves for OS and PFS comparing low (<12 g/ dL for women, <13 g/dL for men) and high (>12 g/dL for women, >13 g/dL for men) Hb levels in the training(a,b), overall(c,d), testing set(e,f), CHOP cohort(g,h) and R-CHOP cohort(i,j). **Figure S6.** Survival curves according to RDW and PLT levels in CHOP cohort and the whole cohort with IPI score 3-5. (1) OS(a,c) and PFS(b,d) according to RDW levels and PLT counts in CHOP cohort with IPI score 3-5. (2) OS(e,g) and PFS(f,h) according to RDW levels and PLT counts in the whole cohort with IPI score 3-5. **Table S1.** Clinical characteristics of healthy donors and DLBCL patients. **Table S2.** Baseline clinical characteristics of patients with DLBCL. **Table S3.** Univariate analysis of clinicopathological parameters for the prediction of OS and PFS in CHOP cohort patients(n=175). **Table S4.** Univariate analysis of clinicopathological parameters for the prediction of OS and PFS in RCHOP cohort patients(n=174).

## Data Availability

The datasets used and analyzed during the current study are available from the corresponding author on a reasonable request.

## Abbreviations

AGR: Albumin/globulin ratio
ALB: Albumin
ALC: Absolute lymphocyte count
AMC: Absolute monocyte count
ANC: Absolute neutrophil count
BM: Bone marrow
CHOP: Cyclophosphamide, doxorubicin, vincristine and prednisone
c-index: Harrell’s concordance index
CNS: Central nervous system
CRP: C-reactive protein
DLBCL: Diffuse large B-cell lymphoma
ECOG PS: Eastern Cooperative Oncology Group performance status
EPO: Erythropoietin
ESR: Erythrocyte sedimentation rate
GLB: Globulin
HB: Hemoglobin
HDs: Healthy donors
HLH: Hemophagocytic lymphohistiocytosis
HRs: Hazard ratios
IPI: International Prognostic Index
LDH: Lactate dehydrogenase
LMR: Lymphocyte/monocyte ratio
NHL: Non-Hodgkin lymphoma
NLR: Neutrophil/lymphocyte ratio
OS: Overall survival
PFS: Progression-free survival
PLT: Platelet count
pPCL: Primary plasma cell leukemia
R-CHOP: Rituximab versus cyclophosphamide, doxorubicin, vincristine and prednisolone
RDW: Red blood distribution width
WBC: White blood cell count
