# Supplementary material for: Red blood cell distribution width and platelet counts are independent prognostic factors and improve the predictive ability of IPI score in diffuse large B-cell lymphoma patients
Source: BMC Cancer. 2019 Nov 11;19:1084. doi: 10.1186/s12885-019-6281-1 (PMC6849243; doi:10.1186/s12885-019-6281-1)
Supplement: Supplementary file 1 — Additional file 1: Figure S1. ROC curves analysis for RDW(a) and PLT(b) in the training set (N=200) of patients with DLBCL. Figure S2. Correlation between RDW, PLT and WBC, NLR, ALB, HB levels in all patients with DLBCL. Correlation between RDW and WBC(a), NLR(b), ALB(c) and HB(d) levels in all patients with DLBCL. (2)Correlation between PLT and WBC(e), NLR(f), ALB(g) and HB(h) levels in all patients with DLBCL. Figure S3. Correlation between HB and WBC(a), NLR(b) and ALB(c) levels in all patients with DLBCL. Figure S4. Survival curves according to RDW and PLT levels in the training, overall and testing set. (1)OS(a,c) and PFS(b,d) according to the RDW and PLT levels in the training set. (2)OS(e,g) and PFS(f,h) according to the RDW and PLT levels in the overall set. (3)OS(i,k) and PFS(j,l) according to the RDW and PLT levels in the testing set. Figure S5. Kaplan–Meier curves for OS and PFS comparing low (<12 g/ dL for women, <13 g/dL for men) and high (>12 g/dL for women, >13 g/dL for men) Hb levels in the training(a,b), overall(c,d), testing set(e,f), CHOP cohort(g,h) and R-CHOP cohort(i,j). Figure S6. Survival curves according to RDW and PLT levels in CHOP cohort and the whole cohort with IPI score 3-5. (1) OS(a,c) and PFS(b,d) according to RDW levels and PLT counts in CHOP cohort with IPI score 3-5. (2) OS(e,g) and PFS(f,h) according to RDW levels and PLT counts in the whole cohort with IPI score 3-5. Table S1. Clinical characteristics of healthy donors and DLBCL patients. Table S2. Baseline clinical characteristics of patients with DLBCL. Table S3. Univariate analysis of clinicopathological parameters for the prediction of OS and PFS in CHOP cohort patients(n=175). Table S4. Univariate analysis of clinicopathological parameters for the prediction of OS and PFS in RCHOP cohort patients(n=174). [file 12885_2019_6281_MOESM1_ESM.doc]

**Red blood cell distribution width and platelet counts are independent prognostic factors and improve the predictive ability of IPI score in diffuse large B-cell lymphoma patients**

Manman Li^1,2#^, Hailong Xia^3#^, Huimin Zheng^1,2#^, Yafeng Li^4^, Jun Liu^1,2^, Linhui Hu^5^, Jingrong Li^6^, Yangyang Ding^1,2^, Lianfang Pu^7^, Qianle Gui^1,2^, Yijie Zheng^8^, Zhimin Zhai^1,2^, Shudao Xiong^1,2^*

*Corresponding author: Shudao Xiong, Department of Hematology/Hematological Lab, The Second Hospital of Anhui Medical University, Hefei 230601, Anhui Province, People’s Republic of China. Email：[xshdao@ahmu.edu.cn](mailto:xshdao@ahmu.edu.cn) Tel：0551-63869344

^#^Co-first authors, the author contributed equally to this work

b

a

**Supplementary Figure 1.** ROC curves analysis for RDW(a) and PLT(b) in the training set (N=200) of patients with DLBCL.

**e**

**f**

**h**

**g**

**d**

**c**

**a**

**b**

**Supplementary Figure 2**. Correlation between RDW, PLT and WBC, NLR, ALB, HB levels in all patients with DLBCL.

1. Correlation between RDW and WBC(a), NLR(b), ALB(c) and HB(d) levels in all patients with DLBCL. (2)Correlation between PLT and WBC(e), NLR(f), ALB(g) and HB(h) levels in all patients with DLBCL.


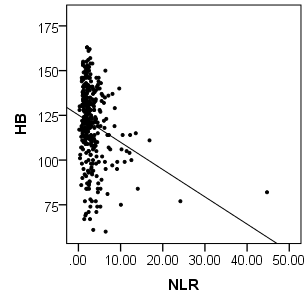


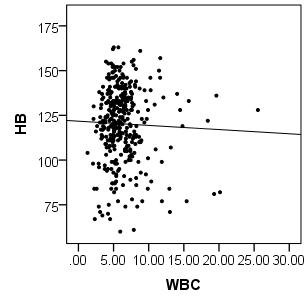


**r=-0.032**

**P=0.547**

**a**

**r=-0.253**

**P＜0.001**

**b**


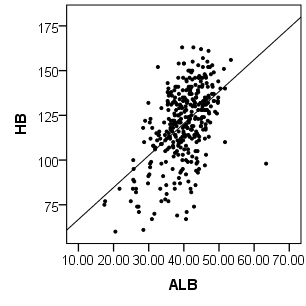


**r=0.519**

**P＜0.001**

**c**

**Supplementary Figure 3**. Correlation between HB and WBC(a), NLR(b) and ALB(c) levels in all patients with DLBCL.


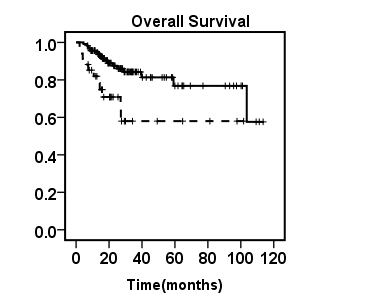


**P=0.005**

**RDW≦14.35**

**RDW>14.35**

**RDW>14.35**

**RDW≦14.35**

**PLT>126.5**

**PLT≦126.5**

**RDW>14.35**

**RDW≦14.35**

**PLT>126.5**

**PLT≦126.5**

**a**

**b**

**P=0.001**

**P=0.074**

**P=0.008**

**P=0.012**

**RDW>14.35**

**RDW≦14.35**

**PLT>126.5**

**PLT≦126.5**

**RDW>14.35**

**RDW≦14.35**

**PLT>126.5**

**PLT≦126.5**

**P=0.006**

**P<0.001**

**P<0.001**

**P=0.003**

**PLT>126.5**

**PLT>126.5**

**PLT≦126.5**

**RDW≦14.35**

**RDW>14.35**

**PLT≦126.5**

**P=0.170**

**P=0.020**

**P=0.035**

**c**

**d**

**e**

**f**

**g**

**h**

**i**

**j**

**k**

**l**

**Supplementary Figure 4**. Survival curves according to RDW and PLT levels in the training, overall and testing set.

(1)OS(a,c) and PFS(b,d) according to the RDW and PLT levels in the training set. (2)OS(e,g) and PFS(f,h) according to the RDW and PLT levels in the overall set. (3)OS(i,k) and PFS(j,l) according to the RDW and PLT levels in the testing set.


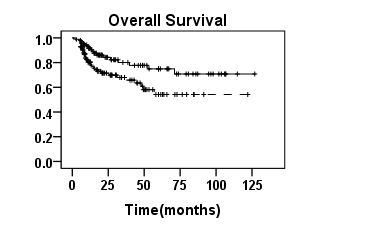

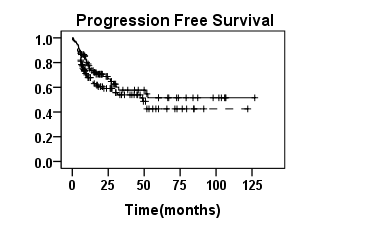

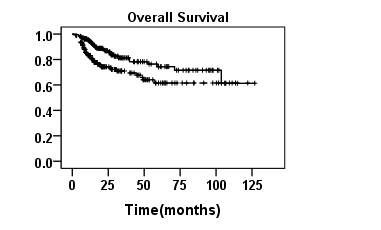

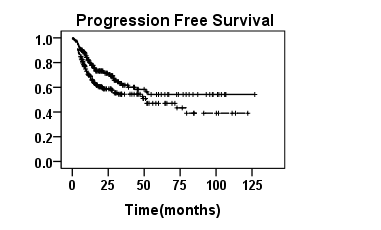

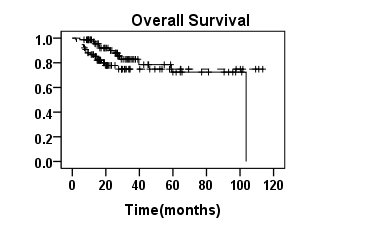

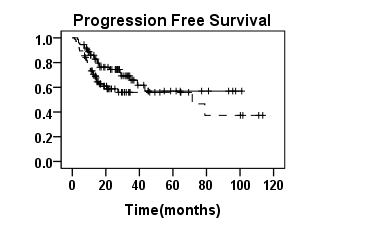

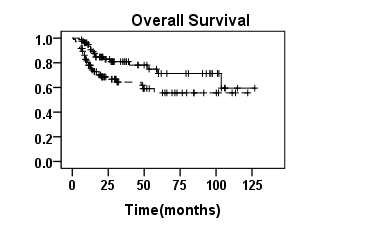

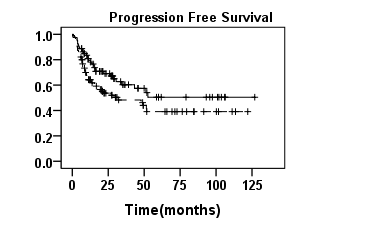

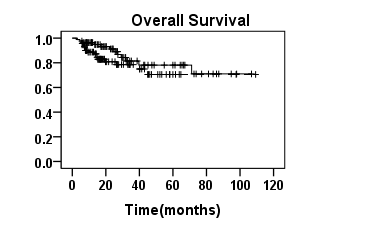

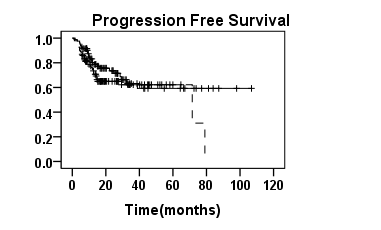


**High Hb levels**

**Low Hb levels**

**High Hb levels**

**Low Hb levels**

**High Hb levels**

**Low Hb levels**

**High Hb levels**

**Low Hb levels**

**High Hb levels**

**Low Hb levels**

**High Hb levels**

**Low Hb levels**

**High Hb levels**

**Low Hb levels**

**High Hb levels**

**Low Hb levels**

**High Hb levels**

**Low Hb levels**

**High Hb levels**

**Low Hb levels**

**P=0.019**

**P=0.182**

**P=0.010**

**P=0.026**

**P=0.313**

**P=0.074**

**P=0.024**

**P=0.060**

**P=0.192**

**P=0.215**

**a**

**b**

**c**

**d**

**e**

**f**

**g**

**h**

**i**

**j**

**Supplementary Figure 5**. Kaplan–Meier curves for OS and PFS comparing low (<12 g/ dL for women, <13 g/dL for men) and high (>12 g/dL for women, >13 g/dL for men) Hb levels in the training(a,b), overall(c,d), testing set(e,f), CHOP cohort(g,h) and R-CHOP cohort(i,j).

**
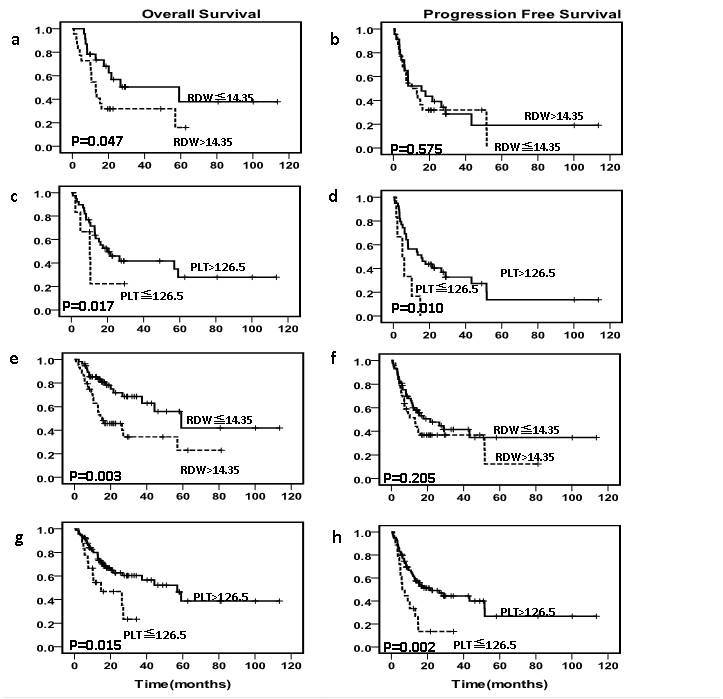
**

**Supplementary Figure 6**. Survival curves according to RDW and PLT levels in CHOP cohort and the whole cohort with IPI score 3-5. (1) OS(a,c) and PFS(b,d) according to RDW levels and PLT counts in CHOP cohort with IPI score 3-5. (2) OS(e,g) and PFS(f,h) according to RDW levels and PLT counts in the whole cohort with IPI score 3-5 .

**Supplementary Table 1. Clinical characteristics of healthy donors and DLBCL patients.**

| characteristics | Number (n, %) or median(range) | | P value |
| --- | --- | --- | --- |
|  | DLBCL Patients(n=349) | Healthy Donors(n=130) |  |
| Age | 55.73(9-89) | 56.48(25-81) | 0.763 |
| Gender (male) | 191(54.73) | 65(50.00) | 0.272 |
| WBC(×10^9^/L) | 6.29(1.3-25.53) | 5.93(3.19-10.1) | 0.832 |
| ANC(×10^9^/L) | 3.96(0.39-18.78) | 3.43(1.79-7.12) | 0.180 |
| ALC(×10^9^/L) | 1.63(0.36-18.51) | 1.94(0.9-3.96) | <0.001 |
| AMC(×10^9^/L) | 0.50(0.04-2.76) | 0.39(0.09-0.72) | 0.001 |
| PLT(×10^9^/L) | 213.7(5-1111) | 205.9(65-439) | 0.348 |
| HB(g/L) | 120.3(60-163) | 142(112-180) | <0.001 |
| RDW(%) | 14.04(11.6-26.5) | 13.14(11.8-19) | <0.001 |
| ALB(g/dL) | 39.9(17.4-63.4) | 43.9(37.2-51.5) | <0.001 |
| GLB(g/dL) | 27.9(14.8-70.7) | 30.5(23.3-37.5) | <0.001 |
| AGR | 1.48(0.45-2.82) | 1.45(1.03-1.96) | 0.402 |

**Supplementary Table 2. Baseline clinical characteristics of patients with DLBCL.**

| Characteristics | Testing set | Training set | P value |
| --- | --- | --- | --- |
| Gender (male) | 81 | 110 | 0.906 |
| Age>60 | 69 | 77 | 0.144 |
| ECOG PS≧2 | 37 | 54 | 0.648 |
| Extranodal sites≧2 | 29 | 57 | 0.053 |
| Ann Arbor stage III/IV | 70 | 104 | 0.354 |
| Serum LDH level≧246u/l | 61 | 85 | 0.770 |
| IPI>2 | 42 | 59 | 0.789 |
| B symptoms(present) | 38 | 65 | 0.156 |
| BM involvement | 6 | 9 | 0.829 |
| Low Hb level | 76 | 111 | 0.405 |
| PLT**≦**126.5 | 14 | 30 | 0.119 |
| RDW>14.35 | 34 | 59 | 0.163 |

**Supplementary Table 3. Univariate analysis of clinicopathological parameters for the prediction of OS and PFS in CHOP cohort patients(n=175).**

| **Parameter** | **Number** | **%** |  | **Overall survival** | | |  | **Progression-free survival** | | |
| --- | --- | --- | --- | --- | --- | --- | --- | --- | --- | --- |
|  |  |  |  | **HR** | **95%CI** | **P value** |  | **HR** | **95%CI** | **P value** |
| Gender(male) | 100 | 57.14 |  | 1.064 | 0.605-1.872 | 0.829 |  | 1.110 | 0.712-1.732 | 0.645 |
| age>60 | 88 | 50.29 |  | 3.732 | 1.939-7.182 | <0.001 |  | 2.689 | 1.710-4.229 | <0.001 |
| PLT≦126.5(×10^9^/L) | 20 | 11.43 |  | 1.519 | 0.710-3.248 | 0.281 |  | 1.533 | 0.827-2.840 | 0.175 |
| RDW>14.35% | 48 | 27.43 |  | 2.736 | 1.554-4.816 | <0.001 |  | 1.701 | 1.066-2.716 | 0.026 |
| Low Hb level | 95 | 54.29 |  | 1.952 | 1.082-3.521 | 0.026 |  | 1.544 | 0.978-2.438 | 0.062 |
| B symptoms(present) | 49 | 28.00 |  | 2.525 | 1.426-4.471 | 0.001 |  | 1.571 | 0.978-2.522 | 0.062 |
| Ann Arbor stage III/IV | 75 | 42.86 |  | 3.619 | 1.988-6.588 | <0.001 |  | 2.689 | 1.710-4.229 | <0.001 |
| ECOG PS>1 | 43 | 24.57 |  | 2.804 | 1.590-4.943 | <0.001 |  | 1.715 | 1.066-2.761 | 0.026 |
| LDH>normal | 65 | 37.14 |  | 2.500 | 1.421-4.397 | 0.001 |  | 1.900 | 1.218-2.964 | 0.005 |
| Extranodal sites>1 | 31 | 17.71 |  | 3.004 | 1.662-5.429 | <0.001 |  | 2.390 | 1.460-3.992 | 0.001 |
| BM involvement * | 7 | 4.00 |  |  |  |  |  |  |  |  |
| IPI>2 | 45 | 25.71 |  | 4.926 | 2.791-8.695 | <0.001 |  | 3.256 | 2.072-5.117 | <0.001 |

***** The bone marrow involvement in univariate analysis did not yield effective results and the number of patients involved in bone marrow was small, it was not included in multivariate analysis.

**Supplementary Table 4. Univariate analysis of clinicopathological parameters for the prediction of OS and PFS in RCHOP** **cohort patients(n=174).**

| **Parameter** | **Number** | **%** |  | **Overall survival** | | |  | **Progression-free survival** | | |
| --- | --- | --- | --- | --- | --- | --- | --- | --- | --- | --- |
|  |  |  |  | **HR** | **95%CI** | **P value** |  | **HR** | **95%CI** | **P value** |
| Gender(male) | 91 | 52.30 |  | 1.215 | 0.589-2.505 | 0.599 |  | 1.037 | 0.613-1.755 | 0.891 |
| age>60 | 58 | 33.33 |  | 2.758 | 1.343-5.666 | 0.006 |  | 1.834 | 0.951-2.808 | 0.076 |
| PLT≦126.5(×10^9^/L) | 24 | 13.80 |  | 3.396 | 1.550-7.440 | 0.002 |  | 3.467 | 1.877-6.402 | <0.001 |
| RDW>14.35% | 45 | 25.86 |  | 2.231 | 1.112-4.842 | 0.025 |  | 1.783 | 1.023-3.107 | 0.041 |
| Low Hb level | 92 | 52.87 |  | 1.627 | 0.777-3.408 | 0.197 |  | 1.400 | 0.821-2.389 | 0.217 |
| B symptoms(present) | 54 | 31.03 |  | 1.773 | 0.850-3.701 | 0.127 |  | 1.741 | 1.013-2.991 | 0.045 |
| Ann Arbor stage III/IV | 99 | 56.90 |  | 2.645 | 1.169-5.985 | 0.020 |  | 2.465 | 1.377-4.413 | 0.002 |
| ECOG PS>1 | 48 | 27.59 |  | 2.690 | 1.295-5.585 | 0.008 |  | 2.264 | 1.316-3.895 | 0.003 |
| LDH>normal | 81 | 46.55 |  | 1.978 | 0.949-4.123 | 0.069 |  | 1.933 | 1.133-3.297 | 0.016 |
| Extranodal sites>1 | 55 | 31.61 |  | 2.394 | 1.165-4.922 | 0.018 |  | 2.566 | 1.510-4.361 | <0.001 |
| BM involvement * | 8 | 4.60 |  |  |  |  |  |  |  |  |
| IPI>2 | 56 | 32.18 |  | 3.286 | 1.587-6.802 | 0.001 |  | 2.292 | 1.348-3.899 | 0.002 |

*****The bone marrow involvement in univariate analysis did not yield effective results and the number of patients involved in bone marrow was small, it was not included in multivariate analysis.
